# Direct Comparison of ^19^F qNMR and ^1^H qNMR by Characterizing Atorvastatin Calcium Content

**DOI:** 10.1155/2016/7627823

**Published:** 2016-09-01

**Authors:** Yang Liu, Zhaoxia Liu, Huaxin Yang, Lan He

**Affiliations:** National Institutes for Food and Drug Control, Beijing 100050, China

## Abstract

Quantitative nuclear magnetic resonance (qNMR) is a powerful tool in measuring drug content because of its high speed, sensitivity, and precision. Most of the reports were based on proton qNMR (^1^H qNMR) and only a few fluorine qNMR (^19^F qNMR) were reported. No research has been conducted to directly compare the advantage and disadvantage between these two methods. In the present study, both ^19^F and ^1^H qNMR were performed to characterize the content of atorvastatin calcium with the same internal standard. Linearity, precision, and results from two methods were compared. Results showed that ^19^F qNMR has similar precision and sensitivity to ^1^H qNMR. Both methods generate similar results compared to mass balance method. Major advantage from ^19^F qNMR is that the analyte signal is with less or no interference from impurities. ^19^F qNMR is an excellent approach to quantify fluorine-containing analytes.

## 1. Introduction

Quantitative nuclear magnetic resonance (qNMR) has been widely utilized in pharmaceutical analysis [1–3], natural products characterization [4, 5], and reference substances quality control [6–9]. This technique has several advantages including fast sample preparation, no necessity for reference material, and generating both structure information and quantification result in one experiment. Currently, most of the qNMR reported are proton qNMR (^1^H qNMR). For some analytes, choosing a suitable internal standard (IS) is often challenging since the response signals in ^1^H NMR are generally from *δ* 0 to 15 and signal overlapping occurs easily. When an analyte is mixed with various excipients in medicines or different metabolites in body fluids, the deployment of ^1^H qNMR might be impossible due to severe signals overlapping. Some groups reported the application of heteronuclear 2D qNMR techniques which can avoid the signal overlapping mentioned previously [10, 11]. But these 2D qNMR are time consuming (more than two hours) and the results are easily affected by T2 and coupling constant which are generally not a crucial parameter in 1D qNMR [10].

^19^F NMR has been utilized in characterizing fluorine-containing pharmaceutical and metabolites in complicated matrices [12, 13] since drug excipients or body fluids barely contain fluorine and do not interfere with analytes. One major advantage of ^19^F NMR is that signals in ^19^F NMR barely overlap due to its broad response range (ca. *δ*  −200 to 100) which makes the selection of IS in ^19^F qNMR much easier than in ^1^H qNMR. Several groups have reported the deployment of ^19^F qNMR in characterizing pharmaceutical [14, 15], metabolites [16], and biooils [17].

Both ^1^H and ^19^F qNMR have their advantages and drawbacks. Although ^1^H qNMR is potentially applicable to any analyte containing proton, the application of this method is limited when analytes are in complicated matrices. And the selection of IS should be careful to avoid interference with the analyte. On the contrary, interference from matrices or IS seldom happens in ^19^F qNMR.

**Figure 1:**
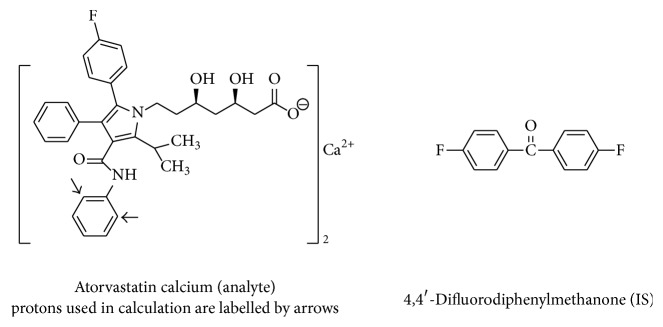
Structures of atorvastatin calcium and 4,4′-difluorodiphenylmethanone.

There is no direct comparison of ^19^F qNMR and ^1^H qNMR such as signal sensitivity, linearity, and RSD. To fully understand the applicable conditions of these two methods, we chose 4,4′-difluorodiphenylmethanone which has both hydrogen and fluorine atoms as an IS to analyze the content of atorvastatin calcium with both ^19^F and ^1^H qNMR (Figure 1). Directly comparing results from the same analyte and IS by different qNMR experiments can avoid the influence of sample purity. Besides, the method validation data from both ^19^F and ^1^H qNMR were also compared.

## 2. Materials and Methods

### 2.1. Materials and Analyte Preparations

4,4′-Difluorodiphenylmethanone was purchased from TCI Chemicals (>99.0%, Shanghai, China); atorvastatin calcium was from Ranbaxy Laboratories (94.7%, Gurgaon, India); and DMSO-*d*_6_ was from Sigma (99.9%, St. Louis, USA). All chemicals were used as received.

4,4′-Difluorodiphenylmethanone and atorvastatin calcium were dissolved in DMSO-*d*_6_ to produce a concentration of about 25 mmol/mL and 8 mmol/mL, respectively.

### 2.2. Instrument Conditions

All of the ^19^F and ^1^H experiments were acquired at 298 K using a Bruker Ascend 500 M spectrometer with a BBO probe at 470.61 MHz and 500.15 MHz, respectively. For ^19^F qNMR, the experiments were under the following parameters: 90° pulse angle, center offset (O1P) = −110 ppm, spectral width (SW) = 100 ppm, 128 K data points, 16 scans, and relaxation time *D*1 = 15 s. For ^1^H-qNMR, the following parameters were applied: 30° pulse angle, O1P = 6.15 ppm, 64 K data points, 16 scans, and relaxation time *D*1 = 15 s.

### 2.3. Processing Parameters

Data was processed on TopSpin 2.1 software with 0.3 Hz exponential apodization applied to FID. Manual phase correction and signal integrations were performed corresponding to the IS signals and analyte signals. ^19^F NMR shift was adjusted with CF_3_COOH  (*δ*  −76.2) and ^1^H NMR shift was referenced to tetramethylsilane.

### 2.4. Content Calculation Formula

The content of analyte was calculated by

(1) $$\begin{array}{c} W_{s}\left(\;\%\;\right)=\frac{\left(A_{s}/n_{s}\right)\times M_{s}\times m_{r}}{\left(A_{r}/n_{r}\right)\times M_{r}\times m_{s}}\times P_{r}\times 100\%, \end{array}$$

where *A*_*s*_ and *A*_*r*_ are the signal response of the analyte and IS, *n*_*s*_ and *n*_*r*_ are the number of spin atoms (fluorine in ^19^F and proton in ^1^H qNMR) in the analyte and IS, *M*_*s*_ is the molecular weight of analyte (1155.4 g/mol), *M*_*r*_ is the molecular weight of IS (218.2 g/mol), *m*_*s*_ and *m*_*r*_ are the mass of the analyte and IS, and *P*_*r*_ is the purity of the IS.

## 3. Result and Discussion

### 3.1. Optimization of Experiment Parameters

Relaxation time (*D*1) is an essential parameter in qNMR experiments. *D*1 should be more than 5 times of longitudinal relaxation (*T*1) to make sure more than 99% of nuclei return to their equilibrium status [18]. *T*1 in ^19^F and ^1^H qNMR experiments were determined by an inversion recovery method. *T*1 of the analyte signals in ^19^F and ^1^H experiments were found to be 0.86 s and 2.18 s, respectively. *T*1 of the IS in ^19^F and ^1^H experiments were 1.80 s and 2.97 s. So *D*1 in both ^19^F and ^1^H qNMR experiments were set as 15 s to make sure the full relaxation is achieved before next repulsion.

Transmitter offset (O1P) and spectral width (SW) are another two important parameters in qNMR experiments. In ^1^H qNMR experiments, default O1P (6.175 ppm) and SW (20 ppm) settings worked well. On the contrary, O1P and SW in ^19^F qNMR experiments must be manually modified. When default O1P (−100 ppm) and SW (241 ppm) were used, the spectrum is difficult to perform phase and baseline correction. In this study, the signals of analyte and IS appeared at *δ*  −111.9 and −104.4, respectively. So O1P was set at the center of two signals *δ*  −108. Meanwhile, it is reported that response signals should not locate at the edge of a spectrum to avoid distortion [19]. Here, SW was set at 60 ppm to fulfill the requirement.

### 3.2. Selection of Analyte Signals and IS Signals

**Figure 2:**
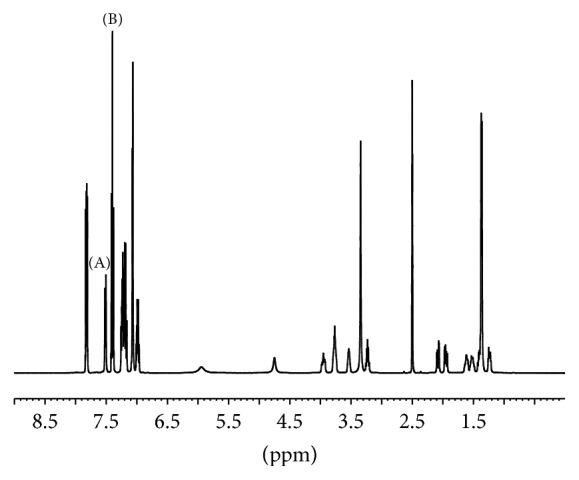
^1^H qNMR spectrum of atorvastatin calcium (A) and internal standard (B).

**Figure 3:**
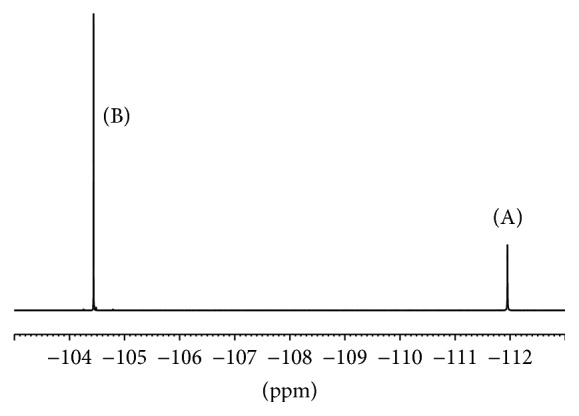
^19^F qNMR spectrum of atorvastatin calcium (A) and internal standard (B).

In ^1^H qNMR experiments, the multiple signals of the analyte are distributed from around *δ* 1.0 to 8.0. Signals at *δ* 7.5 and 7.4 from the analyte and IS, respectively (Figure 2), were chosen for content calculation. Meanwhile, wide response range in a ^19^F qNMR spectrum makes the selection of IS easy and straightforward. Signals overlapping in a ^19^F qNMR experiment rarely occur. Generally, any fluorine-containing compound with high purity which does not react with the analyte is eligible as an IS in ^19^F qNMR experiment. Here, 4,4′-difluorodiphenylmethanone was utilized (Figure 3).

### 3.3. Method Validation

#### 3.3.1. Linearity and Range

^19^F and ^1^H qNMR methods were validated (Table 1). The linearity of both methods was measured by using the solutions prepared by dissolving desired amount of analyte and IS in one tube. The ratio of calculated analyte mass to added analyte mass was fitted to a linear curve. The correction coefficient showed that both ^19^F and ^1^H methods had good linearity within 3.21–20.34 mg/mL concentration ranges with *R* ^2^ > 0.99.

#### 3.3.2. Precision, Repeatability, and Stability

Precision tests were carried out by testing the same solution six times. The repeatability experiments were achieved by characterizing six independent solutions containing both analyte and IS. The RSD of precision and repeatability indicates the good accuracy of the both methods. The stability of solutions was assessed by analyzing one analyte at 1, 2, 4, 6, and 8 hours intervals. The results indicated that both atorvastatin calcium and IS are stable after 8 hours in solution.

#### 3.3.3. Limit Of Quantification (LOQ)

LOQ are calculated as 10*σ*/*S* where *σ* is the standard deviation (SD) of the *Y* intercepts and *S* means the slope of linearity curve [20]. It was found that the LOQ in ^19^F qNMR is similar to that from ^1^H qNMR.

### 3.4. Comparison Results from ^1^H and ^19^F qNMR (Table 2)

Mean of six results from independent solutions was calculated. The content of atorvastatin calcium is 93.1% from ^19^F qNMR and 95.3% from ^1^H qNMR. Both results are consistent with that from mass balance method (94.7%). The major reason that lowers the purity values is water content in analytes. Both ^19^F and ^1^H qNMR can generate accurate results in determining the content of atorvastatin calcium.

## 4. Conclusions

For the first time, ^19^F and ^1^H qNMR were performed and directly compared with the same analyte and IS. Method validation data showed that ^19^F qNMR has similar accuracy, sensitivity, and reproducibility to ^1^H qNMR. The quantitative results from the two methods are comparable to that from mass balance measurement. ^19^F qNMR is valuable in quantitatively analyzing fluorine-containing analytes which are codissolved with excipients, body fluids, or various metabolites. ^19^F qNMR can be widely applied in early drug research and development as well as clinical trials.

## Figures and Tables

**Table 1:** Method validation of ^19^F and ^1^H qNMR measurements.

	^19^F qNMR	^1^H qNMR
Linearity, *r*	0.9999	0.9999
Precision (*n* = 6)		
RSD (%)	0.49	0.82
Repeatability (*n* = 6)		
RSD (%)	0.73	0.62
LOQ (mg/mL)	1.34	1.02

**Table 2:** Content of atorvastatin calcium calculated from ^19^F and ^1^H qNMR measurements.

Number	Atorvastatin calcium content (%)	
	^19^F qNMR	^1^H qNMR
1	93.9	94.8
2	92.2	95.1
3	93.8	95.1
4	93.1	94.9
5	92.8	95.7
6	92.6	96.4

Mean	93.1	95.3
RSD (%)	0.73	0.62
